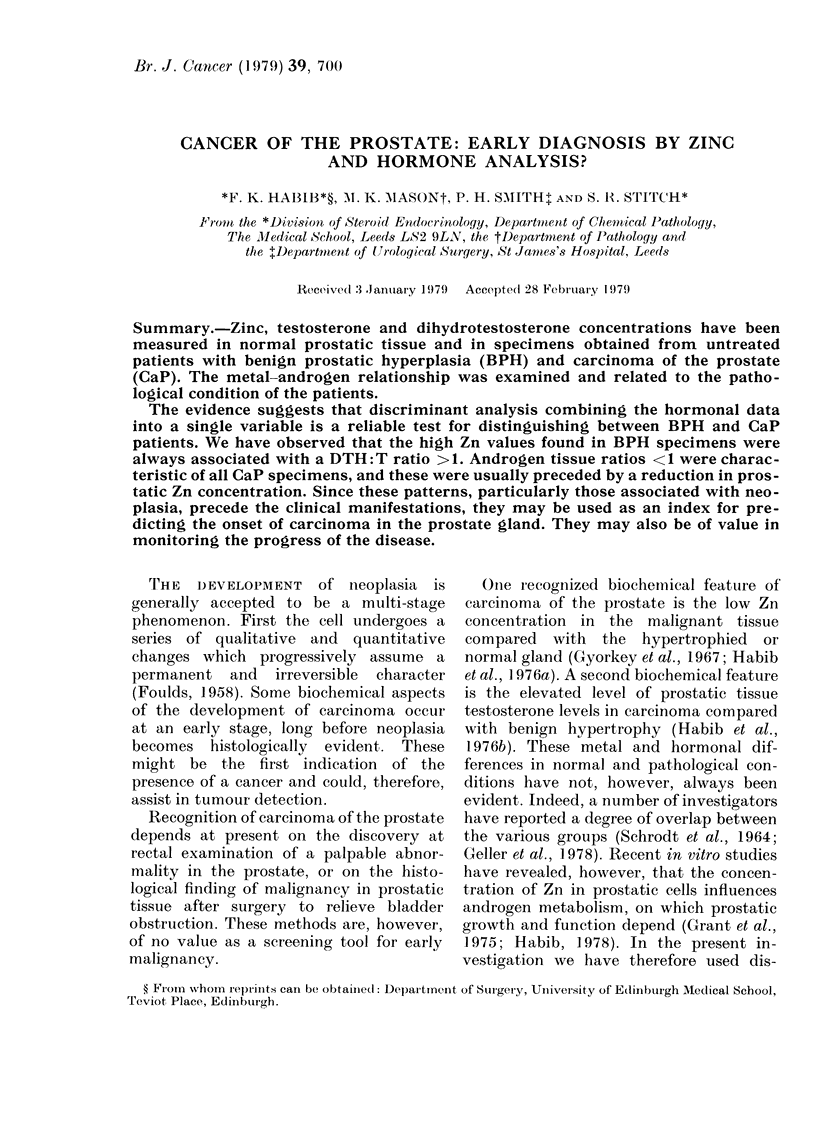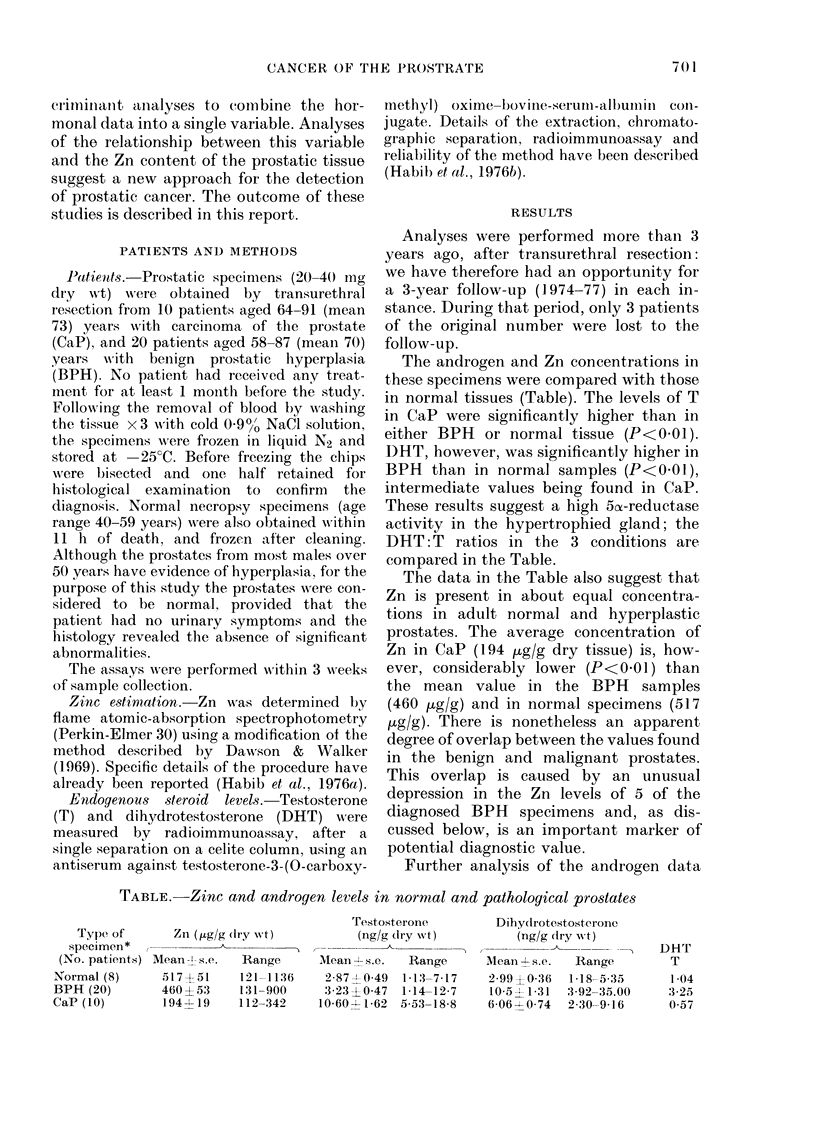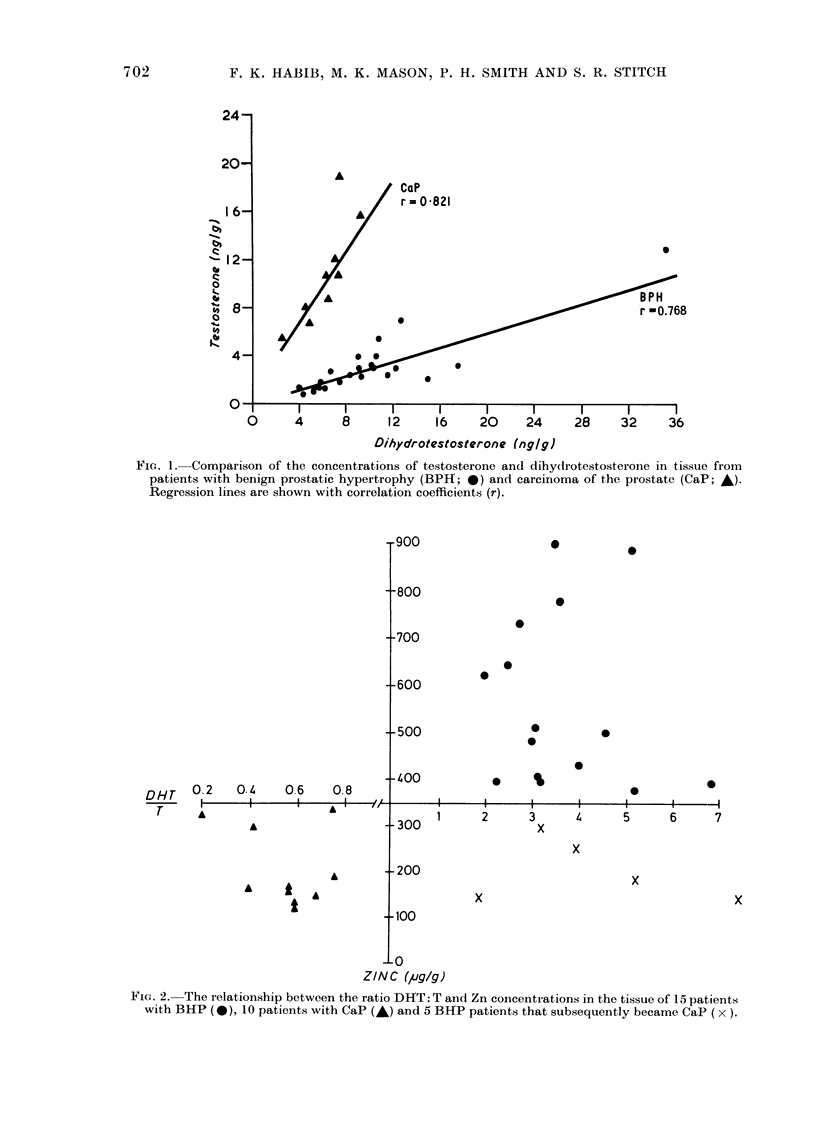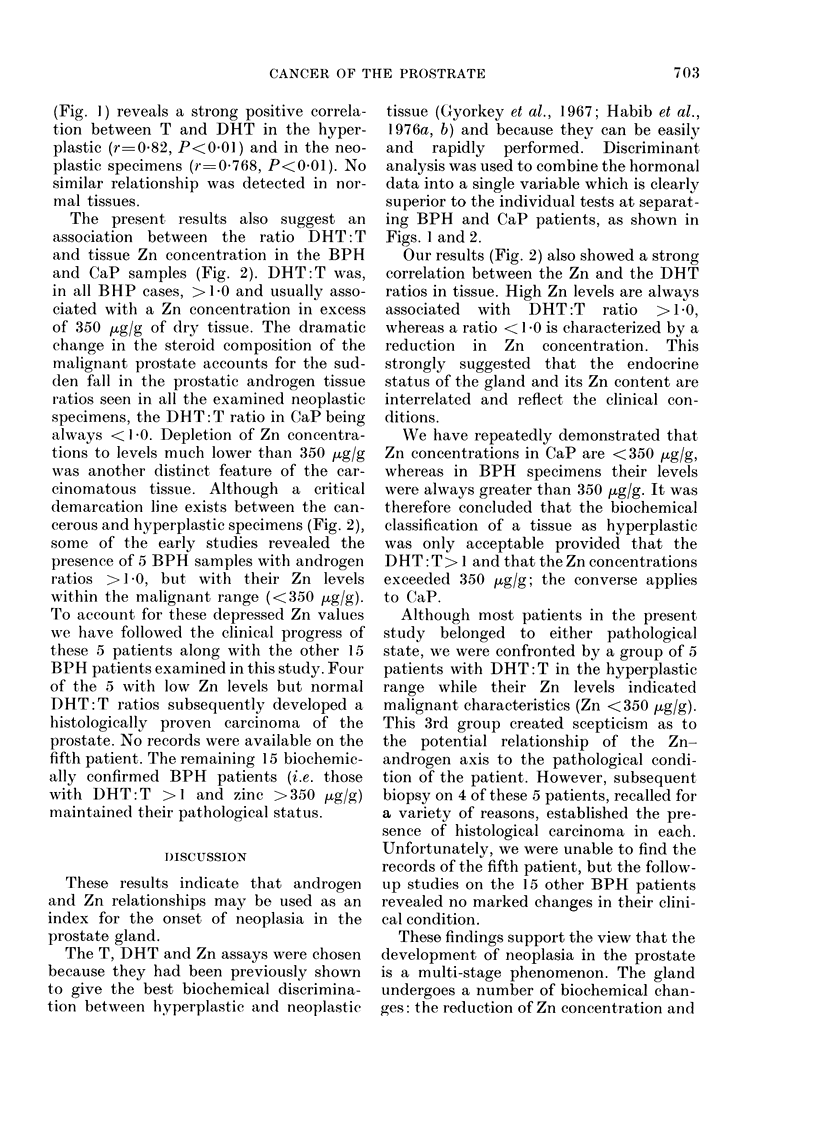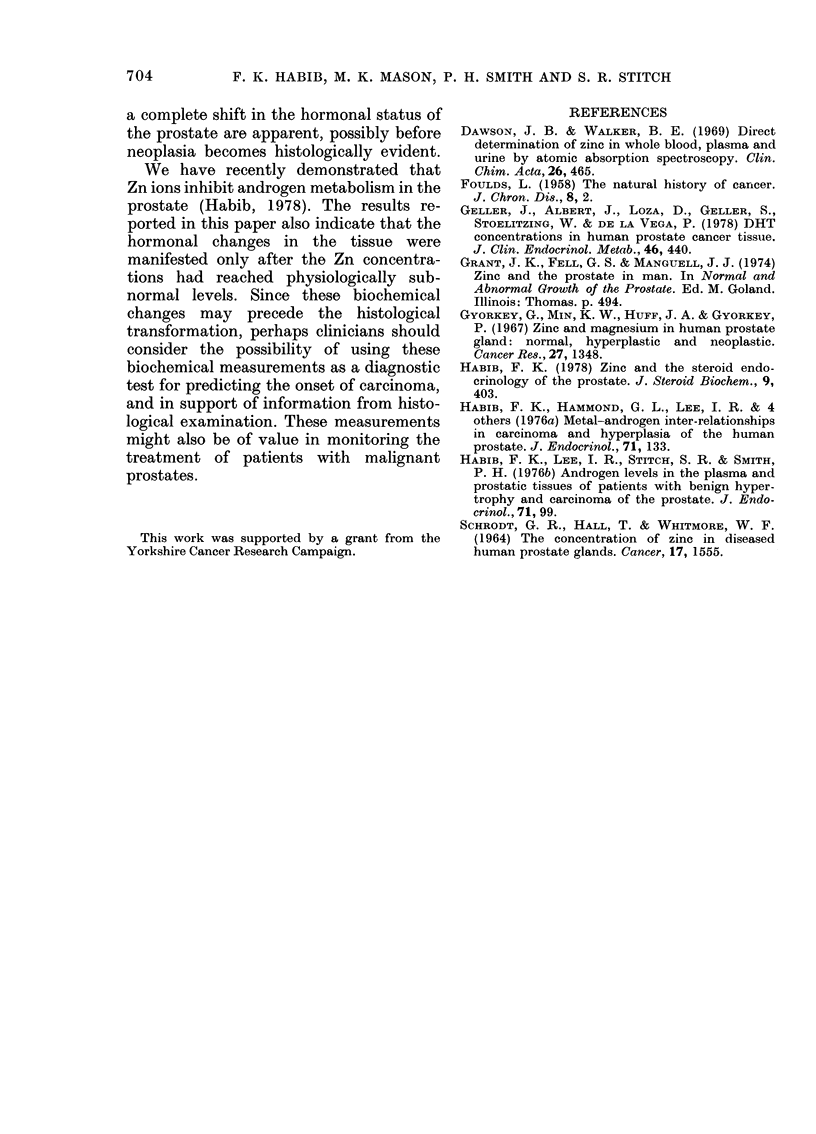# Cancer of the prostate: early diagnosis by zinc and hormone analysis?

**DOI:** 10.1038/bjc.1979.123

**Published:** 1979-06

**Authors:** F. K. Habib, M. K. Mason, P. H. Smith, S. R. Stitch

## Abstract

Zinc, testosterone and dihydrotestosterone concentrations have been measured in normal prostatic tissue and in specimens obtained from untreated patients with benign prostatic hyperplasia (BPH) and carcinoma of the prostate (CaP). The metal--androgen relationship was examined and related to the pathological condition of the patients. The evidence suggests that discriminant analysis combining the hormonal data into a single variable is a reliable test for distinguishing between BPH and CaP patients. We have observed that the high Zn values found in BPH specimens were always associated with a DTH:T ratio greater than 1. Androgen tissue ratios less than 1 were characteristic of all CaP specimens, and these were usually preceded by a reduction in prostatic Zn concentration. Since these patterns, particularly those associated with neoplasia, precede the clinical manifestations, they may be used as an index for predicting the onset of carcinoma in the prostate gland. They may also be of value in monitoring the progress of the disease.


*Br. J. Cancer* (1979) **39**, 700

### CANCER OF THE PROSTATE: EARLY DIAGNOSIS BY ZINC AND HORMONE ANALYSIS?

*F. K. HABIB*§, M. K. MASON†, P. H. SMITH‡ AND S. R. STITCH*

*From the *Division of Steroid Endocrinology, Department of Chemical Pathology, The Medical School, Leeds LS2 9LN, the †Department of Pathology and the ‡Department of Urological Surgery, St James's Hospital, Leeds*

Received 3 January 1979 Accepted 28 February 1979

**Summary.—Zinc, testosterone and dihydrotestosterone concentrations have been measured in normal prostatic tissue and in specimens obtained from untreated patients with benign prostatic hyperplasia (BPH) and carcinoma of the prostate (CaP). The metal–androgen relationship was examined and related to the pathological condition of the patients.**

**The evidence suggests that discriminant analysis combining the hormonal data into a single variable is a reliable test for distinguishing between BPH and CaP patients. We have observed that the high Zn values found in BPH specimens were always associated with a DTH:T ratio >1. Androgen tissue ratios <1 were characteristic of all CaP specimens, and these were usually preceded by a reduction in prostatic Zn concentration. Since these patterns, particularly those associated with neoplasia, precede the clinical manifestations, they may be used as an index for predicting the onset of carcinoma in the prostate gland. They may also be of value in monitoring the progress of the disease.**

THE DEVELOPMENT of neoplasia is generally accepted to be a multi-stage phenomenon. First the cell undergoes a series of qualitative and quantitative changes which progressively assume a permanent and irreversible character (Foulds, 1958). Some biochemical aspects of the development of carcinoma occur at an early stage, long before neoplasia becomes histologically evident. These might be the first indication of the presence of a cancer and could, therefore, assist in tumour detection.

Recognition of carcinoma of the prostate depends at present on the discovery at rectal examination of a palpable abnormality in the prostate, or on the histological finding of malignancy in prostatic tissue after surgery to relieve bladder obstruction. These methods are, however, of no value as a screening tool for early malignancy.

One recognized biochemical feature of carcinoma of the prostate is the low Zn concentration in the malignant tissue compared with the hypertrophied or normal gland (Gyorkey *et al.*, 1967; Habib *et al.*, 1976*a*). A second biochemical feature is the elevated level of prostatic tissue testosterone levels in carcinoma compared with benign hypertrophy (Habib *et al.*, 1976*b*). These metal and hormonal differences in normal and pathological conditions have not, however, always been evident. Indeed, a number of investigators have reported a degree of overlap between the various groups (Schrodt *et al.*, 1964; Geller *et al.*, 1978). Recent *in vitro* studies have revealed, however, that the concentration of Zn in prostatic cells influences androgen metabolism, on which prostatic growth and function depend (Grant *et al.*, 1975; Habib, 1978). In the present investigation we have therefore used dis-

§ From whom reprints can be obtained: Department of Surgery, University of Edinburgh Medical School, Teviot Place, Edinburgh.

criminant analyses to combine the hormonal data into a single variable. Analyses of the relationship between this variable and the Zn content of the prostatic tissue suggest a new approach for the detection of prostatic cancer. The outcome of these studies is described in this report.

### PATIENTS AND METHODS

*Patients.*—Prostatic specimens (20–40 mg dry wt) were obtained by transurethral resection from 10 patients aged 64–91 (mean 73) years with carcinoma of the prostate (CaP), and 20 patients aged 58–87 (mean 70) years with benign prostatic hyperplasia (BPH). No patient had received any treatment for at least 1 month before the study. Following the removal of blood by washing the tissue ×3 with cold 0·9% NaCl solution, the specimens were frozen in liquid $N_2$ and stored at −25°C. Before freezing the chips were bisected and one half retained for histological examination to confirm the diagnosis. Normal necropsy specimens (age range 40–59 years) were also obtained within 11 h of death, and frozen after cleaning. Although the prostates from most males over 50 years have evidence of hyperplasia, for the purpose of this study the prostates were considered to be normal, provided that the patient had no urinary symptoms and the histology revealed the absence of significant abnormalities.

The assays were performed within 3 weeks of sample collection.

*Zinc estimation.*—Zn was determined by flame atomic-absorption spectrophotometry (Perkin-Elmer 30) using a modification of the method described by Dawson & Walker (1969). Specific details of the procedure have already been reported (Habib *et al.*, 1976*a*).

*Endogenous steroid levels.*—Testosterone (T) and dihydrotestosterone (DHT) were measured by radioimmunoassay, after a single separation on a celite column, using an antiserum against testosterone-3-(O-carboxymethyl) oxime–bovine-serum-albumin conjugate. Details of the extraction, chromatographic separation, radioimmunoassay and reliability of the method have been described (Habib *et al.*, 1976*b*).

### RESULTS

Analyses were performed more than 3 years ago, after transurethral resection: we have therefore had an opportunity for a 3-year follow-up (1974–77) in each instance. During that period, only 3 patients of the original number were lost to the follow-up.

The androgen and Zn concentrations in these specimens were compared with those in normal tissues (Table). The levels of T in CaP were significantly higher than in either BPH or normal tissue ($P<0{\cdot}01$). DHT, however, was significantly higher in BPH than in normal samples ($P<0{\cdot}01$), intermediate values being found in CaP. These results suggest a high 5α-reductase activity in the hypertrophied gland; the DHT:T ratios in the 3 conditions are compared in the Table.

The data in the Table also suggest that Zn is present in about equal concentrations in adult normal and hyperplastic prostates. The average concentration of Zn in CaP (194 μg/g dry tissue) is, however, considerably lower ($P<0{\cdot}01$) than the mean value in the BPH samples (460 μg/g) and in normal specimens (517 μg/g). There is nonetheless an apparent degree of overlap between the values found in the benign and malignant prostates. This overlap is caused by an unusual depression in the Zn levels of 5 of the diagnosed BPH specimens and, as discussed below, is an important marker of potential diagnostic value.

Further analysis of the androgen data

TABLE.—*Zinc and androgen levels in normal and pathological prostates*

| Type of specimen* (No. patients) | Zn (μg/g dry wt) | | Testosterone (ng/g dry wt) | | Dihydrotestosterone (ng/g dry wt) | | $\frac{DHT}{T}$ |
|---|---|---|---|---|---|---|---|
| | Mean ± s.e. | Range | Mean ± s.e. | Range | Mean ± s.e. | Range | |
| Normal (8) | 517 ± 51 | 121–1136 | 2·87 ± 0·49 | 1·13–7·17 | 2·99 ± 0·36 | 1·18–5·35 | 1·04 |
| BPH (20) | 460 ± 53 | 131–900 | 3·23 ± 0·47 | 1·14–12·7 | 10·5 ± 1·31 | 3·92–35.00 | 3·25 |
| CaP (10) | 194 ± 19 | 112–342 | 10·60 ± 1·62 | 5·53–18·8 | 6·06 ± 0·74 | 2·30–9·16 | 0·57 |

FIG. 1.—Comparison of the concentrations of testosterone and dihydrotestosterone in tissue from patients with benign prostatic hypertrophy (BPH; ●) and carcinoma of the prostate (CaP; ▲). Regression lines are shown with correlation coefficients (*r*).

FIG. 2.—The relationship between the ratio DHT:T and Zn concentrations in the tissue of 15 patients with BHP (●), 10 patients with CaP (▲) and 5 BHP patients that subsequently became CaP (×).

(Fig. 1) reveals a strong positive correlation between T and DHT in the hyperplastic ($r=0{\cdot}82$, $P<0{\cdot}01$) and in the neoplastic specimens ($r=0{\cdot}768$, $P<0{\cdot}01$). No similar relationship was detected in normal tissues.

The present results also suggest an association between the ratio DHT:T and tissue Zn concentration in the BPH and CaP samples (Fig. 2). DHT:T was, in all BHP cases, $>1{\cdot}0$ and usually associated with a Zn concentration in excess of 350 $\mu$g/g of dry tissue. The dramatic change in the steroid composition of the malignant prostate accounts for the sudden fall in the prostatic androgen tissue ratios seen in all the examined neoplastic specimens, the DHT:T ratio in CaP being always $<1{\cdot}0$. Depletion of Zn concentrations to levels much lower than 350 $\mu$g/g was another distinct feature of the carcinomatous tissue. Although a critical demarcation line exists between the cancerous and hyperplastic specimens (Fig. 2), some of the early studies revealed the presence of 5 BPH samples with androgen ratios $>1{\cdot}0$, but with their Zn levels within the malignant range ($<350$ $\mu$g/g). To account for these depressed Zn values we have followed the clinical progress of these 5 patients along with the other 15 BPH patients examined in this study. Four of the 5 with low Zn levels but normal DHT:T ratios subsequently developed a histologically proven carcinoma of the prostate. No records were available on the fifth patient. The remaining 15 biochemically confirmed BPH patients (*i.e.* those with DHT:T $>1$ and zinc $>350$ $\mu$g/g) maintained their pathological status.

### DISCUSSION

These results indicate that androgen and Zn relationships may be used as an index for the onset of neoplasia in the prostate gland.

The T, DHT and Zn assays were chosen because they had been previously shown to give the best biochemical discrimination between hyperplastic and neoplastic tissue (Gyorkey *et al.*, 1967; Habib *et al.*, 1976*a*, *b*) and because they can be easily and rapidly performed. Discriminant analysis was used to combine the hormonal data into a single variable which is clearly superior to the individual tests at separating BPH and CaP patients, as shown in Figs. 1 and 2.

Our results (Fig. 2) also showed a strong correlation between the Zn and the DHT ratios in tissue. High Zn levels are always associated with DHT:T ratio $>1{\cdot}0$, whereas a ratio $<1{\cdot}0$ is characterized by a reduction in Zn concentration. This strongly suggested that the endocrine status of the gland and its Zn content are interrelated and reflect the clinical conditions.

We have repeatedly demonstrated that Zn concentrations in CaP are $<350$ $\mu$g/g, whereas in BPH specimens their levels were always greater than 350 $\mu$g/g. It was therefore concluded that the biochemical classification of a tissue as hyperplastic was only acceptable provided that the DHT:T$>1$ and that the Zn concentrations exceeded 350 $\mu$g/g; the converse applies to CaP.

Although most patients in the present study belonged to either pathological state, we were confronted by a group of 5 patients with DHT:T in the hyperplastic range while their Zn levels indicated malignant characteristics (Zn $<350$ $\mu$g/g). This 3rd group created scepticism as to the potential relationship of the Zn–androgen axis to the pathological condition of the patient. However, subsequent biopsy on 4 of these 5 patients, recalled for a variety of reasons, established the presence of histological carcinoma in each. Unfortunately, we were unable to find the records of the fifth patient, but the follow-up studies on the 15 other BPH patients revealed no marked changes in their clinical condition.

These findings support the view that the development of neoplasia in the prostate is a multi-stage phenomenon. The gland undergoes a number of biochemical changes: the reduction of Zn concentration and a complete shift in the hormonal status of the prostate are apparent, possibly before neoplasia becomes histologically evident.

We have recently demonstrated that Zn ions inhibit androgen metabolism in the prostate (Habib, 1978). The results reported in this paper also indicate that the hormonal changes in the tissue were manifested only after the Zn concentrations had reached physiologically subnormal levels. Since these biochemical changes may precede the histological transformation, perhaps clinicians should consider the possibility of using these biochemical measurements as a diagnostic test for predicting the onset of carcinoma, and in support of information from histological examination. These measurements might also be of value in monitoring the treatment of patients with malignant prostates.

This work was supported by a grant from the Yorkshire Cancer Research Campaign.

### REFERENCES

DAWSON, J. B. & WALKER, B. E. (1969) Direct determination of zinc in whole blood, plasma and urine by atomic absorption spectroscopy. *Clin. Chim. Acta*, **26**, 465.

FOULDS, L. (1958) The natural history of cancer. *J. Chron. Dis.*, **8**, 2.

GELLER, J., ALBERT, J., LOZA, D., GELLER, S., STOELITZING, W. & DE LA VEGA, P. (1978) DHT concentrations in human prostate cancer tissue. *J. Clin. Endocrinol. Metab.*, **46**, 440.

GRANT, J. K., FELL, G. S. & MANGUELL, J. J. (1974) Zinc and the prostate in man. In *Normal and Abnormal Growth of the Prostate*. Ed. M. Goland. Illinois: Thomas. p. 494.

GYORKEY, G., MIN, K. W., HUFF, J. A. & GYORKEY, P. (1967) Zinc and magnesium in human prostate gland: normal, hyperplastic and neoplastic. *Cancer Res.*, **27**, 1348.

HABIB, F. K. (1978) Zinc and the steroid endocrinology of the prostate. *J. Steroid Biochem.*, **9**, 403.

HABIB, F. K., HAMMOND, G. L., LEE, I. R. & 4 others (1976*a*) Metal–androgen inter-relationships in carcinoma and hyperplasia of the human prostate. *J. Endocrinol.*, **71**, 133.

HABIB, F. K., LEE, I. R., STITCH, S. R. & SMITH, P. H. (1976*b*) Androgen levels in the plasma and prostatic tissues of patients with benign hypertrophy and carcinoma of the prostate. *J. Endocrinol.*, **71**, 99.

SCHRODT, G. R., HALL, T. & WHITMORE, W. F. (1964) The concentration of zinc in diseased human prostate glands. *Cancer*, **17**, 1555.